# Influence of socioeconomic status on cognitive outcome after childhood arterial ischemic stroke

**DOI:** 10.1111/dmcn.14779

**Published:** 2020-12-18

**Authors:** Lisa Bartha‐Doering, Andreas Gleiss, Sarah Knaus, Maria Theresa Schmook, Rainer Seidl

**Affiliations:** ^1^ Department of Pediatrics and Adolescent Medicine Medical University of Vienna Vienna Austria; ^2^ Comprehensive Centre for Pediatrics Medical University of Vienna Vienna Austria; ^3^ Centre for Medical Statistics, Informatics, and Intelligent Systems Medical University of Vienna Vienna Austria; ^4^ Department of Biomedical Imaging and Image‐guided Therapy Medical University of Vienna Vienna Austria

## Abstract

**Aim:**

To determine whether socioeconomic status (SES) is a stronger predictor for cognitive outcome after childhood arterial ischemic stroke compared to clinical factors.

**Method:**

We investigated perceptual reasoning, executive functions, language, memory, and attention in 18 children and adolescents (12 males, six females, median age at testing 13y 4mo, range 7y–17y 5mo) after arterial ischemic stroke; collected sociodemographic information (education of parents, household income); and used clinical information (initial lesion volume, residual lesion volume, age at stroke, time since stroke). Linear regression models were used to investigate the potential influence of SES and clinical parameters on cognitive abilities.

**Results:**

SES had a moderate effect on all cognitive outcome parameters except attention by explaining 41.9%, 37.9%, 38.0%, and 22.5% of variability in perceptual reasoning, executive functions, language, and memory respectively. Initial lesion volume was the only clinical parameter that showed moderate importance on cognitive outcome (33.1% and 25.6% of the variability in perceptual reasoning and memory respectively). Overall, SES was a stronger predictor of cognitive outcome than clinical factors.

**Interpretation:**

Future paediatric studies aiming at clinical predictors of cognitive outcome should control their analyses for SES in their study participants. The findings of the present study further point to the need for more attention to the treatment of children with low SES.

**What this paper adds:**

- Socioeconomic status (SES) explains up to 42% of variance in cognitive outcome after childhood arterial ischemic stroke.
- SES is a stronger predictor of outcome than clinical factors.

## What this paper adds

- Socioeconomic status (SES) explains up to 42% of variance in cognitive outcome after childhood arterial ischemic stroke.
- SES is a stronger predictor of outcome than clinical factors.

**Abbreviations**
modASPECTS: Modified paediatric version of the Alberta Stroke Program Early Computed Tomography Score
SES: Socioeconomic status
WWT: Wortschatz‐ und Wortfindungstest

Across educational and income groups, children do not have the same opportunities when growing up. The relationship between socioeconomic status (SES) and health in children has been well documented over many years. This substantial relationship begins before birth, when children from low SES families are more likely to show growth restriction and neurological deficits in utero, and proceeds throughout childhood into adolescence, where low SES is associated with a higher risk of injury and is implicated in a number of diseases.^1^ Moreover, the SES of children is significantly associated with cognitive development. Several studies have identified low parental education and low family income as important predictors of later IQ and academic achievement.^2^

When children experience health problems, the consequences are often more pronounced in those with lower SES. SES is associated with long‐term health consequences in preterm birth, infections throughout childhood, and child injury, among others.^3^ Similarily, in childhood stroke, SES has shown to have some influence on neurological outcome.^4^ A comprehensive multicentre study (Vascular Effects of Infection in Pediatric Stroke [VIPS] study) with 355 children after stroke found that very low household income was associated with worse outcome using the Pediatric Stroke Outcome Measure, a standard neurological measure examining mental status; cranial nerves; motor, sensory, and cerebellar functions; and gait.^5^ Maternal education level or type of residence (rural, urban) were not associated with neurological outcome in this study.

Large prospective studies were conducted to evaluate neurological outcome after stroke, including the international VIPS study described above,^5^ a Swiss study,^6^ a Canadian study,^7^ and a UK study.^8^ Three of these studies did not formally assess cognition, and markers of SES were not collected in the last two studies. However, half of the children who experience a stroke face residual cognitive impairment. Smaller studies have demonstrated that, when investigated in detail, executive function, processing speed, attention, language, and memory are most often affected after childhood stroke.^9^, ^10^ While some studies have shown that age at stroke, lesion size, and lesion location have some influence on cognitive outcome, overall clinical factors do not explain much of the variance of cognitive abilities after stroke.^6^, ^10^, ^11^ This raises the question about the influence of SES on the cognitive outcome in these children.

The aim of the present study was to determine whether SES is a stronger predictor for cognitive outcome after childhood arterial ischemic stroke compared to clinical factors. We examined a relatively small but homogeneous group of children and administered a comprehensive neuropsychological test battery to evaluate various cognitive domains. Our a priori hypothesis was that SES would explain more variance of cognitive outcome than clinical factors.

## METHOD

### Participants

Eighteen children and adolescents (12 males, six females) with unilateral arterial ischemic stroke were recruited between 2014 and 2017 at the neuropediatric outpatient unit of the Department of Pediatrics and Adolescent Medicine, Medical University of Vienna, Austria. Inclusion criteria for patients were a single arterial ischemic stroke in one hemisphere, radiological evidence of an ischemic stroke, native German speaking, normal hearing, and normal or corrected‐to‐normal vision. Exclusion criteria were an active seizure disorder, antiseizure medication, or developmental problems before stroke. Table 1 displays the patient characteristics. This observational study was approved by the Ethics Committee of the Medical University of Vienna and conducted in accordance with the Helsinki Declaration of 1975. For children, age‐appropriate assent forms were provided; parents received a parental permission form. All children and one parent per child gave written informed consent before inclusion.

**Table 1:** Clinical and demographic information

	*n*	Median	25th	75th	Range
Sex, female/male	6/12				
Side of stroke, left/right	9/9				
*Stroke location:*					
Cortical	1				
Subcortical	5				
Combined cortical+subcortical	12				
Age at stroke, y:mo		9:7	6:1	12:5	0:1–16:7
Age at testing, y:mo		13:4	9:10	15:6	7:0–17:5
Time between stroke and testing, y:mo		1:8	0:7	7:1	0:2–10:2
Handedness (–1.0 to +1.0)		0.9	0.6	1.0	–1.0 to 1.0
ModASPECTS (1–30)		4.0	2.0	8.0	1–11
Residual lesion volume, cm^3^		17.3	0.7	90.2	0.9–331.5
Maternal education (5‐point scale)		2.0	1.0	4.0	1–5
Paternal education (5‐point scale)		2.0	1.8	3.3	1–5
Household income (6‐point scale)		4.5	2.0	5.3	1–6

modASPECTS, modified paediatric version of the Alberta Stroke Program Early Computed Tomography Score.

### Neuroimaging

3D structural magnetic resonance imaging (MRI) was performed using an isocubic magnetization‐prepared rapid gradient echo sequence (T1‐weighted, TE/TR_4.21/2300ms, inversion time 900ms, with a matrix size of 240×256×160mm, voxel size 1×1×1.10mm, flip angle 9°) sequence on a 3T Siemens TIM Trio scanner (Siemens, Erlangen, Germany). MRIcron was used to define the lesions in the T1‐weighted images.^12^ For each patient, lesions were outlined manually on axial slices on the T1‐weighted images and were then normalized to the Montreal Neurological Institute space with the clinical toolbox for SPM12 (Wellcome Department of Cognitive Neurology, London, UK) implemented in MATLAB 2019a (Version 9.6, Mathworks, Inc., Sherborn, MA, USA). Lesion volumes in cm^3^ were calculated, applying MRIcron's descriptive statistics option on the normalized volumes of interest.

### Retrospective analysis of initial MRI

The modified paediatric version of the Alberta Stroke Program Early Computed Tomography Score (modASPECTS)^13^ was used to retrospectively assess initial infarct volume on axial diffusion‐weighted MRI in all supratentorial regions, including the territories of the anterior, middle, and posterior cerebral arteries as well as the thalamus. A region was scored positive if it was involved in the stroke area, yielding a maximum modASPECTS of 30.

### SES

Educational levels of parents and household income were used as indicators of the children’s SES. Information was obtained in a semi‐structured interview with one parent. The educational levels of the parents were rated on a 5‐point scale for the mother and the father separately: (1) secondary school, (2) apprenticeship, (3) vocational school, (4) school leaving examination (general qualification for university entrance), and (5) university degree. The family household yearly gross net income was classified on a 6‐point scale where higher scores reflect higher income (ranging from less than 10 000€, 10 000–19 000€, 20 000–29 000€, 30 000–39 000€, 40 000–49 000€, and 50 000€ and above).

### Neuropsychological assessment

Perceptual reasoning was evaluated using the three subtests block design, matrix reasoning, and picture completion of the Hamburg‐Wechsler Intelligenztest für Kinder.^14^ These subtests measure visual perception, organization, and reasoning with visually presented, nonverbal material. Executive functioning involving task switching, planning, problem‐solving, and verbal flexibility was tested by the Trail Making Test subtest B,^15^ the Tower of London,^16^ and the Regensburger Wortflüssigkeitstest.^17^ Language tests comprised different tests of vocabulary, comprehension, and reception of grammar, and were examined using the Wortschatz‐ und Wortfindungstest (WWT),^18^ the TROG‐D,^19^ the German version of the Test for Reception of Grammar,^20^ and the Token Test for Children.^21^ Memory was tested for visual and verbal‐auditory stimuli separately using the Rey‐Osterrieth Complex Figure Test^22^ and the German version of the Auditory Verbal Learning Test,^23^ the Verbaler Lern‐ und Merkfähigkeitstest.^24^ Attention and concentration performance was evaluated using the d2 Test of Attention.^25^ Poststroke handedness was evaluated using the Edinburgh Handedness Inventory.^26^

### Statistical analyses

The SES was calculated by taking the arithmetic mean of maternal education (5‐point scale), paternal education (5‐point scale), and household income (rescaled from 6‐point scale to 5‐point scale as: [income–1]*4/5+1). Raw scores of cognitive tests were transformed into age‐adjusted percentiles for each test. Because of missing normative data, WWT raw scores of the children aged 11 years and above were transformed into percentiles based on the norms of children aged 10 years 11 months. Based on previous literature,^27^ we clustered the test scores into six cognitive domains by calculating, if necessary, means of the corresponding subtest percentile ranks.

Because of non‐normal distributions, clinical and demographic characteristics as well as percentile ranks of cognitive tests are presented using median, quartiles, and range. In order to investigate the potential influence of SES and clinical parameters on cognitive test percentiles linear regression models were used. Each predictor’s effect on a cognitive outcome was estimated in a simple linear regression and quantified by its regression coefficient with 95% confidence interval (CI). Because of right‐skewed distributions, residual lesion volume and time since stroke were log‐transformed with log‐basis 1.2 such that regression coefficients quantify a 20% increase in the predictor. Model residuals were checked for approximate normal distribution using histograms and Q–Q plots. The marginal *R* ^2^ value quantifies the proportion of variation in the cognitive outcome that is explained by the predictor. It can thus be interpreted as the importance of the factor in predicting the outcome. Using multiple linear regression models, each clinical predictor’s effect was adjusted for SES. Adjusted regression coefficients are reported with 95% CIs to quantify the clinical predictor’s effect while keeping SES constant. The partial *R* ^2^ value equals the proportion of variation that is explained by a clinical predictor in addition to what is already explained by SES.

Model estimation was performed using SAS 9.4 (SAS Institute, Cary, NC, USA). Descriptive statistics were produced using SPSS Statistics (Version 26; IBM Corp., Armonk, NY, USA). *P*‐values below 0.05 indicated statistical significance. The potential influence of multiple predictors on multiple outcomes was tested in a series of regression models as described above. However, since this is an exploratory study, no correction for multiple testing was performed; the reported uncorrected *p*‐values and CIs have to be interpreted accordingly.

## RESULTS

### Cognitive test results

Group results of cognitive tests are presented in Table 2 (for individual test results, please see Appendix S1 and Fig. S1, online supporting information). Median cognitive performances were within the normal range, with notably lower median scores in verbal fluency, attention, syntactic comprehension, and both verbal and visual memory. The score ranges point to a large heterogeneity of cognitive performances in the study group.

**Table 2:** Group percentile ranks of cognitive tests

	Median	25th	75th	Range
*Perceptual reasoning*	39.5	6.4	61.8	0.3–91.9
Spatial visualization (HAWIK block design)	50.0	8.0	63.0	1.0–99.0
Visual organization (HAWIK picture completion)	37.0	8.0	50.0	1.0–84.0
Visual reasoning (HAWIK matrix reasoning)	37.0	21.0	63.0	2.0–75.0
*Executive functions*	33.6	23.6	47.4	4.0–73.5
Verbal fluency (RWT)	12.0	4.3	27.4	2.5–68.5
Task switching (TMT)	55.0	25.0	63.0	1.0–84.0
Problem‐solving (TL)	61.5	16.3	91.8	5.0–99.0
*Language*	55.5	14.4	67.0	2.0–95.3
Expressive vocabulary (WWT)	67.5	0.0	67.5	0.0–96.0
Language comprehension (Token Test)	65.0	34.0	74.5	5.0–97.0
Syntactic comprehension (Trog‐D)	28.0	9.5	64.0	0.0–97.0
*Memory*	28.1	19.1	43.3	6.5–79.7
Verbal short‐ and long‐term memory (VLMT)	30.0	13.3	43.3	5.3–86.7
Visual short‐ and long‐term memory (RCTF)	29.0	14.0	41.5	5.3–65.3
*Attention (d2)*	25.5	10.5	64.0	3.0–88.0

Raw scores of cognitive tests were transformed into age‐adjusted percentiles. Percentile ranks between 15.9–84.1 are considered to lie within the average range (SD –1.0 to 1.0) of normative data. HAWIK, Hamburg‐Wechsler Intelligenztest für Kinder; RCTF, Rey‐Osterrieth Complex Figure Test; RWT, Regensburger Wortflüssigkeitstest; TL, Tower of London; TMT, Trail Making Test; Trog‐D, Test for Reception of Grammar (German version); VLMT, Verbaler Lern‐ und Merkfähigkeitstest; WWT, Wortschatz‐ und Wortfindungstest.

### Predictors of cognitive outcome in patients with paediatric stroke

The SES exhibited a moderate influence (i.e. marginal *R* ^2^ above approximately 0.25) on all cognitive outcome measures except attention: this factor explained 41.9%, 37.9%, 38.0%, and 22.5% of the variability in perceptual reasoning, executive functions, language, and memory respectively (see Table 3 for the details; see Fig. S2, online supporting information, for graphical presentation). One additional point in the SES of the child added 15.64 more per‐percentile points to the perceptual reasoning score (*p*=0.004), 14.36 more percentile points to the language score (*p*=0.007), 9.58 more percentile points to the executive score (*p*=0.007), and 6.95 more percentile points to the memory score (*p*=0.047) respectively. In contrast, the modASPECTS was the only one of the investigated clinical parameters showing moderate importance for two of the outcomes, for perceptual reasoning (33.1% of variability, –5.31 percentile points for each additional point in the modASPECTS, *p*=0.012) and memory (25.6% of variability, –2.83 percentile points for each additional point in the modASPECTS, *p*=0.032).

**Table 3:** Predictors of cognitive outcome in childhood stroke

Outcome	Predictor	Unadjusted					Adjusted for SES				
		Regression coefficient	95% CI		*p*	Marginal *R* ^2^	Regression coefficient	95% CI		*p*	Partial *R* ^2^
Perceptual reasoning	SES (+1 score point)	**15.64**	**5.89**	**25.40**	**0.004**	**0.419**					
	Age at stroke ( +1y)	2.09	–1.28	5.47	0.207	0.097	1.99	–0.60	4.58	0.122	0.088
	modASPECTS (+1 score point)	**–5.31**	**–9.31**	**–1.31**	**0.012**	**0.331**	**–3.96**	**–7.34**	**–0.57**	**0.025**	**0.170**
	Residual lesion volume (+20%)	–0.89	–1.84	0.07	0.066	0.195	**–0.83**	**–1.54**	**–0.12**	**0.024**	**0.171**
	Time since stroke (+20%)	–0.71	–2.86	1.44	0.494	0.030	–0.36	–2.09	1.38	0.668	0.007
Executive function	SES	**9.58**	**3.07**	**16.08**	**0.007**	**0.379**					
	Age at stroke	0.04	–2.25	2.33	0.973	0.000	–0.02	–1.90	1.85	0.978	0.000
	modASPECTS	0.12	–3.03	3.27	0.939	0.000	1.23	–1.37	3.82	0.331	0.039
	Residual lesion volume	0.29	–0.37	0.96	0.365	0.052	0.33	–0.20	0.86	0.206	0.065
	Time since stroke	–0.49	–1.88	0.89	0.460	0.035	–0.28	–1.43	0.88	0.616	0.011
Language	SES	**14.36**	**4.63**	**24.09**	**0.007**	**0.380**					
	Age at stroke	2.78	–0.32	5.87	0.075	0.185	**2.69**	**0.31**	**5.07**	**0.030**	**0.173**
	modASPECTS	–3.45	–7.80	0.90	0.112	0.150	–2.09	–5.93	1.76	0.266	0.051
	Residual lesion volume	–0.70	–1.66	0.26	0.141	0.131	–0.65	–1.41	0.11	0.090	0.112
	Time since stroke	–0.31	–2.41	1.79	0.756	0.006	0.02	–1.72	1.76	0.981	0.000
Memory	SES	**6.95**	**0.11**	**13.79**	**0.047**	**0.225**					
	Age at stroke	1.33	–0.71	3.37	0.185	0.107	1.29	–0.55	3.13	0.157	0.100
	modASPECTS	**–2.83**	**–5.39**	**–0.27**	**0.032**	**0.256**	–2.27	–4.80	0.26	0.076	0.152
	Residual lesion volume	–0.54	–1.12	0.04	0.064	0.198	**–0.52**	**–1.04**	**–0.00**	**0.050**	**0.180**
	Time since stroke	–0.15	–1.47	1.18	0.817	0.003	0.01	–1.21	1.24	0.981	0.000
Attention	SES	0.30	–13.3	13.88	0.963	0.000					
	Age at stroke	–0.35	–3.85	3.15	0.834	0.003	–0.36	–4.02	3.31	0.837	0.003
	modASPECTS	–1.21	–6.19	3.76	0.609	0.019	–1.31	–6.79	4.17	0.615	0.020
	Residual lesion volume	–0.13	–1.23	0.96	0.797	0.005	–0.13	–1.28	1.01	0.806	0.005
	Time since stroke	1.34	–0.88	3.57	0.217	0.107	1.41	–0.96	3.77	0.221	0.113

In each model, unadjusted and adjusted, 18 observations were used. Regression coefficients quantify the effect on the respective outcome (in percentile points) resulting from a change in the predictor as reported in brackets (multiplicative for residual lesion volume and time since stroke due to logarithmic transformation). Significant findings (*p*≤0.05) are indicated in bold type. CI, confidence interval; modASPECTS, modified paediatric version of the Alberta Stroke Program Early Computed Tomography Score; SES, socioeconomic status.

Partial *R* ^2^ values in Table 3 indicate whether a clinical parameter had importance for predicting an outcome that was beyond that of the SES. This was the case in three outcome measures, where clinical factors had some low importance (i.e. partial *R* ^2^ between approximately 0.10 and 0.25). While the SES explained 41.9% of the variability in perceptual reasoning, the modASPECTS added 17.0%, and log of residual lesion volume added 17.1%. Keeping the SES constant (e.g. by comparing two patients with the same SES) an increase by one score point in the modASPECTS lowered perceptual reasoning by 3.96 percentile points on average (*p*=0.025), whereas a 20% increase in residual lesion volume lowered it by 0.83 percentile points on average (*p*=0.024). For memory, the SES explained 22.5% of variability, the log of residual lesion volume added 18.0% (*p*=0.05 ). Finally, in language functions, the SES explained 38.0% of the variability, while the age at stroke explained an additional 17.3% (*p*=0.030). Keeping constant SES, each additional year of age at stroke occurrence increased language scores by 2.69 percentile points. Overall, SES was a stronger predictor than clinical factors in our study group.

## DISCUSSION

This study investigated cognitive functions in 18 children and adolescents after arterial ischemic stroke, collected sociodemographic information through a semi‐structured interview, and used clinical information to determine whether SES is a stronger predictor for the cognitive outcome compared to clinical factors. We found that SES had a moderate effect on all cognitive outcome parameters except attention by explaining up to 42% of variance in data and was a stronger predictor of cognitive outcome than clinical factors.

### SES as a predictor of cognitive outcome

Why does SES explain nearly half of the variance of cognitive outcome after stroke? We assume four main reasons for this finding. First, the financial situation in families may influence availability, frequency, and duration of treatment, as well as the organization of additional learning support for children after stroke. In Austria, diagnostic care is free of charge for children and adolescents until the end of education and is covered by statutory health insurance. Therapeutic care, however, is a little more complex. Only a limited number of physiotherapy, speech and language therapy, and occupational therapy sessions are covered by health insurance, and therapists working on a health insurance contract are often fully booked and have long waiting lists. Neurocognitive training offered by neuropsychologists is not covered by health insurance at all. In rural areas, availability of therapy is further reduced. Parents with higher incomes thus often finance therapy sessions in private practice or pay for additional sessions to increase therapy frequency and/or duration. The organization of transport to therapy and adult supervision during sessions may also be more difficult for families with lower incomes. Moreover, additional learning support, including tutors, extra educational resources, and learning aids, is not covered by statutory health insurance. Thus, children from families with lower incomes may not get the same quality and quantity of therapy and learning support compared to children with higher SES.

Second, parental education may be associated with the child’s cognitive reserve and, thus, influence cognitive outcome after stroke. Cognitive reserve refers to individual differences in the neural reorganization of cognitive processing and focuses on the functionality of brain processes.^28^ After traumatic brain injury, cognitive reserve has been shown to be a moderator of responsiveness to neuropsychological intervention for adolescents.^29^ In population norm adolescents and adults, cognitive reserve is significantly associated with premorbid intelligence measures,^30^ whereas in children, where premorbid cognitive measures are not quantifiable or not easy to interpret, parental intelligence has been taken as a surrogate for the child’s cognitive reserve.^31^, ^32^ We assume that the same genetic and environmental factors leading to higher educational levels in parents may also impact the cognitive reserve in children.^33^ As a consequence, children with increased flexibility in the reorganization of cognitive functions may have a better chance for favourable cognitive outcome after stroke.^10^

Third, not all children in this study experienced cognitive deficits after stroke, and findings may therefore also reflect the relationship between SES and cognitive functioning in typically developing children.^34^ In Appendix S1, we describe an additional investigation of the effect of SES in a typically developing control group. We found that each additional point in the SES of a typically developing child added 9.3 more percentile points to the perceptual reasoning score, compared to a 15.6 percentile point average increase of perceptual reasoning rank score per additional SES unit in the stroke group. This effect of SES on cognition was not significantly different between groups. Thus, in both groups, parents with higher levels of education may enable better childhood experiences and educational environment, resulting in improved cognitive performance.

Lastly, in previous studies, lower SES was not only associated with neurological outcome, but also with a higher incidence of childhood arterial ischemic stroke.^5^ Could this association add to the worse cognitive outcome in children with low SES? Paediatric stroke is less influenced by typical adult risk factors including diabetes mellitus, arteriosclerosis, hypertension, or smoking. However, a decreased parental level of consciousness at stroke presentation has been reported to be associated with an increased risk of poor neurological outcome.^4^ The authors hypothesized that in lower‐income families, more severe stroke symptoms might be necessary for parents to seek medical help. Furthermore, worse nutritional status and increased childhood obesity associated with lower income^35^ might influence childhood stroke incidence, and, subsequently, the course of the disease. However, further studies are needed to disentangle this complex relationship.

### Clinical predictors of cognitive outcome

Besides SES, we found a moderate effect of the initial infarct volume on memory and perceptual reasoning in our study group, and when SES was kept constant, the initial infarction volume explained an additional 15% and 17% of variance respectively. This is in line with previous studies that have identified an association between neurological outcome and initial infarct volume in children after stroke.^36^ Infarct location, seizures at stroke onset, and stroke recurrence have also been associated with neurological stroke outcome in previous studies.^8^, ^37^ Unfortunately, the present study cannot add information about the predictive value of these factors, as the sample size did not allow us to test infarct location as a possible predictor. Moreover both seizures and stroke recurrence were among the exclusion criteria to avoid too much heterogeneity in our small study sample.

Nevertheless, with SES kept constant we found two further clinical parameters that explained some additional variance. First, the residual lesion volume at the time of cognitive testing accounted for 17% and 18% of variance in perceptual reasoning and memory respectively. Residual lesion volume is most likely associated with initial lesion volume; thus, this result corresponds to the previously reported moderate impact of initial lesion volume on cognitive outcome. Second, the age at stroke explained an additional 17% of variance in language, with older age at stroke being associated with better language abilities when SES was kept constant. This topic has been controversially discussed in the literature and findings so far have been contradictory. While some studies showed better cognitive outcomes in perinatal stroke compared to childhood stroke,^38^ the majority reported worse outcomes in stroke acquired during the perinatal phase compared to later onset.^39^, ^40^, ^41^, ^42^ Nevertheless, the present study did not include children who had experienced perinatal stroke, and previous studies focusing on childhood stroke alone did not find an association between age at stroke and later language functioning.^10^, ^43^, ^44^, ^45^, ^46^ However, compared to age at stroke, SES is a far better predictor of language outcome, explaining 38% of variance in language outcome in the present study – meaning 13 percentile points of language score more per life year at stroke occurrence. As previous studies did not collect markers of SES, this may have skewed their analyses of clinical predictors.

### Limitations

Some limitations of our study have to be considered. First, because of the rarity of disease and the strict inclusion criteria for study participants, the patient group is relatively small. Because of the low sample size, we must expect the power to be low, meaning that non‐significant results have a high chance of being false negative. In addition, *R* ^2^ values exhibit large variability. Thus, generalizability of our results to future patients is limited, although there is a clear qualitative difference between SES and clinical parameters regarding importance as predictors of outcome.

Second, the period between stroke and examination shows large variability. Thus, in some children, reorganizational processes may not have been completed. Though the post‐stroke period did not predict any of the outcome parameters, it probably had an influence on individual results.

Third, because of the lack of normative data of the expressive vocabulary test WWT for children at and older than 12 years of age, we transformed their WWT raw scores based on the norms of the children aged 10 years 11 months. The mean difficulty to name the items of the WWT decreases exponentially and phases out in a flat curve at 10 years of age;^34^ nevertheless, we may have overestimated the expressive vocabulary scores in the older children.

Fourth, two children with left‐side stroke suffered from right motor dysfunctions at the time of testing. These children had been right‐handed premorbidly but used their left hand for writing and drawing in the examination. Both children had normal motor speed in the tests. One child, however, exhibited constructional deficits in copying a complex figure; the other performed within the lower average range. Though most errors could be attributed to planning deficits and detail presence, thus pointing to higher visuo‐constructional deficits, detail inaccuracies also occurred in both children. Thus, left hand clumsiness may have influenced the results of some cognitive tests in these two children.

### Implications of findings

Our study suggests that SES is a stronger predictor for cognitive outcome after childhood arterial ischemic stroke compared to clinical factors. Thus, we propose that future paediatric studies of clinical predictors of cognitive outcome should control their analyses for SES in their study participants.

Above all, however, these findings point to the need for more attention to the treatment of children with low SES. Increased funding and resources should be made available for low SES families. Access to treatment programmes, including neurocognitive training, physiotherapy, speech and language therapy, and occupational therapy, should be guaranteed to children from families with low SES, and transportation to and from therapy sessions should be free of charge for them. High frequency treatment should be offered especially in the early period after stroke, and treatment programmes and recurrent rehabilitation phases should be planned throughout childhood and adolescence. Furthermore, schoolteachers and tutors should be sensitized and trained to improve teaching and learning for this particularly vulnerable patient group, help to create an information‐rich environment for the child, build a learning community, and foster parental involvement in learning.

## Supporting information


**Appendix S1:** Individual test analyses and results.

**Figure S1:** Individual cognitive profiles in patients after stroke.

**Figure S2:** Predictors of cognitive outcome in childhood stroke.

## Data Availability

The data that support the findings of this study are available from the corresponding author upon reasonable request.
