# Supplementary material for: Influence of socioeconomic status on cognitive outcome after childhood arterial ischemic stroke
Source: Dev Med Child Neurol. 2020 Dec 18;63(4):465–71. doi: 10.1111/dmcn.14779 (PMC7986130; doi:10.1111/dmcn.14779)
Supplement: Supplementary file 1 — Appendix S1: Individual test analyses and results. [file DMCN-63-465-s002.docx]

**Appendix S1**

**Individual test analyses**

In line with clinical conventions, individual test scores from SD –1.0 to SD 1.0 (percentile ranks 15.9-84.1) were defined within the average range. Performance below SD –1.0 was read as below average, and performance below SD –2.0 (percentile rank 2.3) was interpreted as impaired.

**Individual test results**

In the individual analyses, 4 out of 18 stroke patients (22%) exhibited impairment (SD -2.0) in one or more cognitive domains (Figure Supplement). These children presented impaired perceptual reasoning; one of them furthermore showed impairment in the overall language score. All these children showed additional cognitive domain scores below average (SD –1.0). Six further stroke patients (33%) revealed one or more cognitive domains below average, with attention and language being affected most often. Individual test scores were missing for attention in two children, and for verbal memory, visual memory, and syntactic comprehension in one child each.

Thus, when investigated in detail, 56% of children with stroke (10/18) displayed below average or impaired cognitive functions, the majority of them (6) in multiple cognitive domains.
