# Supplementary material for: Influence of socioeconomic status on cognitive outcome after childhood arterial ischemic stroke
Source: Dev Med Child Neurol. 2020 Dec 18;63(4):465–71. doi: 10.1111/dmcn.14779 (PMC7986130; doi:10.1111/dmcn.14779)
Supplement: Supplementary file 2 — Figure S1: Individual cognitive profiles in patients after stroke. [file DMCN-63-465-s001.docx]

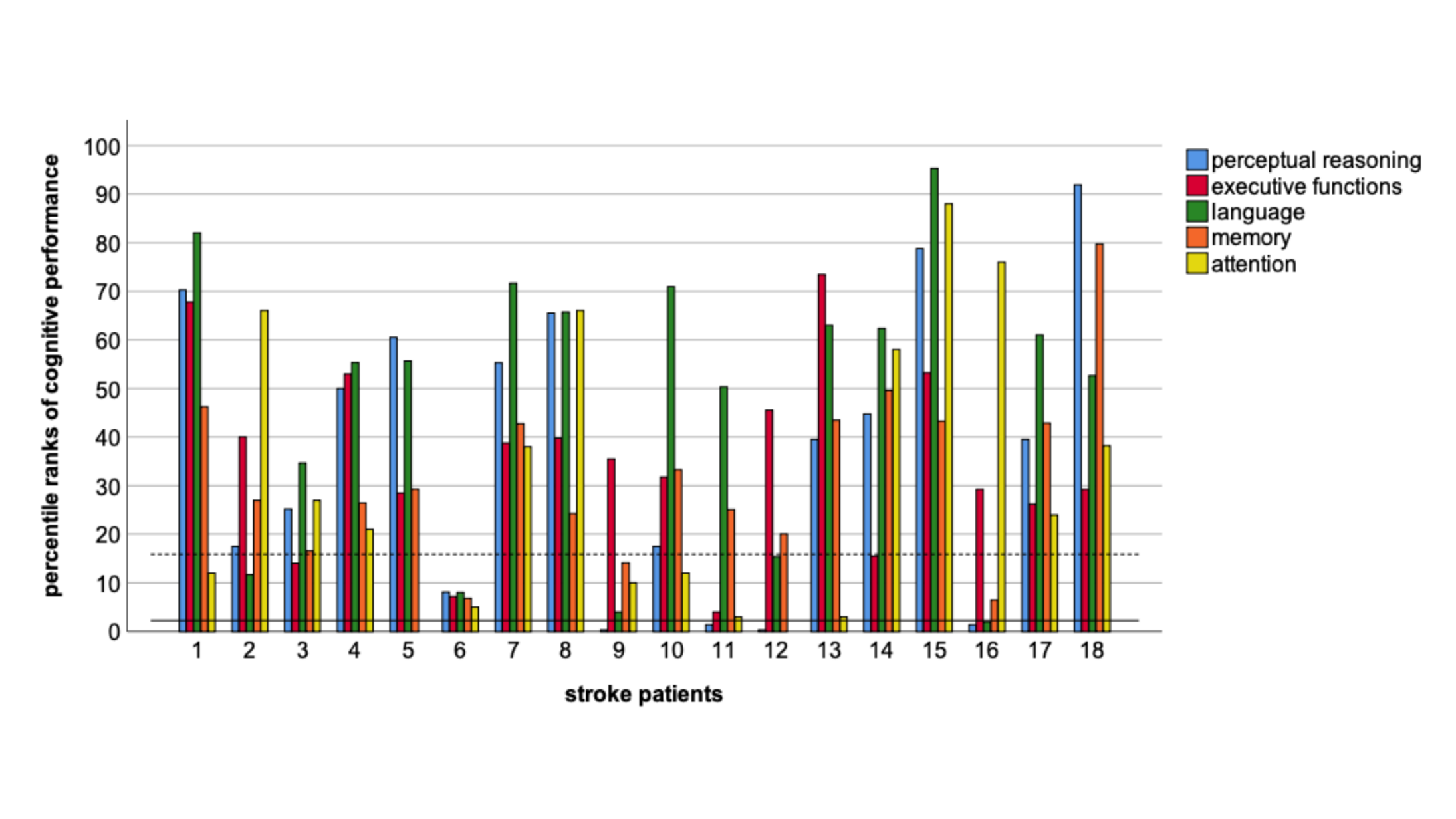


Figure S1. Individual cognitive profiles in stroke patients. The solid line represents SD –2.0 (impaired function), the dashed line SD –1.0 (below average function).
