# Supplementary material for: Influence of socioeconomic status on cognitive outcome after childhood arterial ischemic stroke
Source: Dev Med Child Neurol. 2020 Dec 18;63(4):465–71. doi: 10.1111/dmcn.14779 (PMC7986130; doi:10.1111/dmcn.14779)
Supplement: Supplementary file 3 — Figure S2: Predictors of cognitive outcome in childhood stroke. [file DMCN-63-465-s003.docx]

**Graphical presentation of predictors of cognitive outcome in childhood stroke**


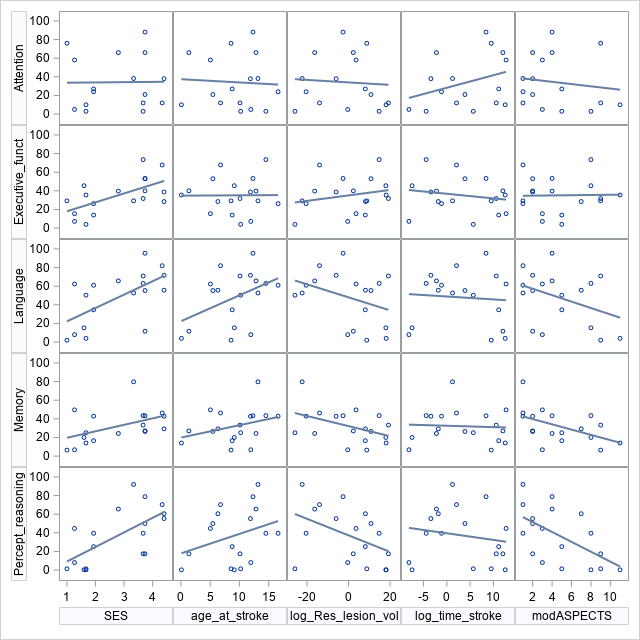


Figure S2. Predictors of cognitive outcome in childhood stroke. Executive_funct = executive functioning; log_time_stroke = log-transformed time since stroke; log_Res_Lesion_vol = log-transformed residual lesion volume; modASPACTS = modified pediatric version of the Alberta Stroke Program Early Computed Tomography Score; Percept_reasoning = perceptual reasoning; SES = socioeconomic status.
